# Gut Microbiota Composition of Biliary Atresia Patients Before Kasai Portoenterostomy Associates With Long-term Outcome

**DOI:** 10.1097/MPG.0000000000003234

**Published:** 2021-07-14

**Authors:** Daan van Wessel, Mark Nomden, Janneke Bruggink, Ruben de Kleine, Alexander Kurilshikov, Henkjan Verkade, Hermie Harmsen, Jan Hulscher

**Affiliations:** ∗ Division of Pediatric Surgery, Department of Surgery; † Division of Pediatric Gastroenterology and Hepatology, Department of Pediatrics; ‡ Division of Hepatico-Pancreatico-Biliary Surgery and Liver Transplantation, Department of Surgery; § Department of Genetics; || Department of Medical Microbiology, University of Groningen, University Medical Center Groningen, The Netherlands.

**Keywords:** biliary atresia, cholestatic liver disease, clearance of jaundice, gut microbiota, gut-liver axis, Kasai portoenterostomy

## Abstract

**Background and Aims:**

Biliary atresia (BA) is a cholestatic, fibro-obliterative cholangiopathy of unknown etiology. BA is primarily treated by a surgical approach, that is, the Kasai portoenterostomy (KPE), to obtain clearance of jaundice (COJ). The gut microbiota (GM) composition has been associated with the course of several cholestatic liver diseases. It is largely unknown, however, whether GM composition associates with the outcome of KPE. We compared the GM composition of BA patients and controls and assessed if GM composition before KPE was related to COJ after KPE.

**Methods:**

We compared feces of term-born BA patients before KPE and controls (patients undergoing inguinal hernia repair) by 16S rRNA sequencing. Composition and alpha diversity of the GM were compared between BA and controls before KPE and after KPE, between patients with COJ versus without COJ (total serum bilirubin < or ≥20 μmol/L <6 months post-KPE).

**Results:**

Alpha diversity was comparable between BA (n = 12, age 1.6 [1.3–1.8] months) and controls (n = 6, age 2.0 [1.4–2.1] months; *P* = 0.22). Compared with controls, BA patients had lower abundances of Bifidobacteriaceae (β = −1.98, *P* < 0.001) and Lachnospiraceae (β = −1.84, *P* = 0.007), and higher abundances of *Streptococcus* (β = −1.13, *P* = 0.003). The alpha diversity before KPE correlated negatively with COJ (*R* = −0.63, *P* = 0.03). Lower alpha diversity pre-KPE was associated with COJ [+] (β_logit_ = −0.64, *P* = 0.04). We observed greater abundances of genus *Acinetobacter* (β = 1.27, *P* = 0.03) and family *Clostridiaceae* (β = 1.45, *P* = 0.03) and lower abundances of the family Enterobacteriaceae (genera *Klebsiella* (β = −1.21, *P* = 0.01), *Salmonella* (β = −1.57, *P* = 0.02)) in COJ [+] versus COJ [−].

**Conclusions:**

The GM of BA patients before Kasai portoenterostomy associates with outcome, clearance of jaundice, suggestive of predictive, and mechanistic roles of the gut microbiota composition in bile homeostasis.

**What Is Known/What Is New**

**What Is Known**

- The gut microbiota composition is associated with the presence of several cholestatic liver diseases.
- Clearance of jaundice is an important predictor of long-term outcome in biliary atresia.
- Predictors of long-term outcome before Kasai portoenterostomy are lacking.

**What Is New**

- The composition of the gut microbiota of patients with biliary atresia differs from that of controls.
- The composition of the gut microbiota before Kasai portoenterostomy associates with clearance of jaundice.
- The gut microbiota composition in biliary atresia patients may hold predictive and mechanistic roles in bile homeostasis.

Biliary atresia (BA) is a rare, progressive obliterative cholangiopathy of infancy, presenting as neonatal cholestasis. Its cause has remained largely unknown. Primary treatment consists of the Kasai hepatoportoenterostomy (KPE). The KPE is associated with long-term survival with native liver in case clearance of jaundice (COJ) is achieved [defined as a total serum bilirubin (TSB) <20 μmol/L (1)] within 6 months after KPE. TSB levels post-KPE, and more recently serum bile acids, are regarded important surrogate markers for long-term outcome (1,2). A successful KPE might avert, yet more frequently delays, liver transplantation (LTx) in BA. At least two-thirds of all BA patients require LTx before reaching adulthood, of which the majority before the age of 1 year (1,3).

It is increasingly recognized that the composition of the gut microbiota (GM) is associated with the presence and course of liver disease, as well as with bile acid homeostasis (4,5). Cirrhosis is associated with a disrupted composition of the GM, as well as with increased gut wall permeability (6–8). The latter allows pathological bacterial translation (ie, increased translocation of bacteria and/or their products, such as lipopolysaccharide [LPS]) from the gut towards the mesenteric lymphatic system, portal vein, and eventually the liver (6). These associations indicate that the ‘gut-liver-axis’ holds important, yet not completely unraveled roles in health and disease. An improved understanding of this gut-liver axis is likely to provide better insights into pathophysiological mechanisms of liver diseases (9). Only a limited number of studies on GM in BA have been published. Data suggest that disturbed bile flow affects the GM composition and, reciprocally, that the GM composition may affect the success rate of KPE surgery (10–12). As bile is absent in BA patients, the composition of their GM can be used as a model to investigate the effect of the absence and subsequent restoration of bile flow on GM composition. To further increase the understanding of the mutual interactions in the gut-liver axis, we aimed to determine in more detail the relationship between the composition of the GM in BA patients and controls, and the possible association between the presurgical GM composition and bile flow after surgery.

## PATIENTS AND METHODS

### Ethics and Patient Inclusion

This prospective pilot study was conducted conforming the guidelines of the Institutional Review Board of the University Medical Center Groningen (UMCG, METc 2015/261) and the 1975 Declaration of Helsinki. All Dutch BA patients are referred to the UMCG for Kasai portoenterostomy (KPE). Patients with type 3 BA (ie, the most common subtype, >90% of cases, obliteration of the proximal part of the extrahepatic biliary tree within the porta hepatis) that underwent KPE were candidates for the present study. Exclusion criteria were primary liver transplantation (LTx), a BA subtype other than type 3, and preterm birth (<37 weeks of gestation). As controls, we included term-born, age-matched patients without comorbidities and use of medication at time of admission or previous use of antibiotics, that underwent inguinal hernia repair. Informed consent was obtained for every included patient.

All BA patients received amoxicillin-clavulanic acid peri-KPE and started a 6-month trimethoprim/sulfamethoxazole oral prophylactic regimen thereafter (dosage 120 mg, bd). None received steroids. Ursodeoxycholic acid (UDCA) therapy was initiated as soon as the stools consistently regained color (stool color card ≥4) (13). All patients received fat-soluble vitamin supplementation and were fed with medium chain triglyceride (MCT)-enriched formula, or breast milk with MCT supplementation. Included control patients did not receive any antibiotic treatment perioperatively. Controls were fed formula or breast milk.

### Patient Data and Clinical Endpoint

For patient demographics, perioperative and clinical follow-up data, we used the Netherlands Study group on Biliary Atresia Registry (NeSBAR) database. We analyzed the age at KPE, feeding method, degree of urbanicity using area address density, postoperative use of antibiotics and UDCA, and biochemistry. Conventional biochemistry data were retrieved from patient charts. In addition to these, we performed Enzyme-linked immunosorbent assays (ELISA, Supplementary material 1) on parameters associated with intestinal integrity and bacterial translocation. Levels of intestinal fatty acid-binding protein (IFABP, enterocyte damage), claudin-3 (cell-cell adhesion), lipopolysaccharide (LPS, major component of outer membrane of Gram-negative bacteria), lipopolysaccharide-binding protein (LBP, binds LPS and presents LPS to soluble cluster of differentiation 14), soluble cluster of differentiation 14 (sCD-14, required for LPS/LBP recognition by toll-like receptor 4 [TLR4] to induce pro-inflammatory cytokines and type 1 interferons) and interleukin-6 (IL-6, released by Kupffer cells upon stimulation by, eg, LPS) were analyzed.

Clearance of jaundice [COJ, total serum bilirubin <20 μmol/L within 6 months after KPE (1)] was used to categorize the KPE as successful. Follow-up ended at last known visit, LTx or death. Sample collection stopped at these points in time.

### Sample Collection

We collected serum and fecal samples from BA patients and controls, immediately at first presentation in our center, before any dietary or medical intervention. Fecal samples were additionally collected at 1 week and at 1, 3, and 6 months post-KPE in BA patients, if available from the patient's diapers. This was either done in an outpatient or clinical setting, depending on the patient's status. After KPE patients received feeding and medical interventions as earlier described. Collection time of day was not standardized because of logistical reasons. Fecal samples were immediately stored at −20 °C and shortly thereafter transferred to −80 °C until further analysis. Whole blood samples were centrifuged and supernatant was collected. Supernatant was stored at −80 °C until further analysis. Samples were taken in singular. Tests were performed in a sterile environment.

### Fecal DNA Extraction, MiSeq Preparation, and Sequence Reads Analysis

DNA was extracted from 0.25 g feces, as previously shown (14). Modified 341F and 806R primers (Supplementary material 2), including a 6-nucleotide sample specific barcode and flow cell adaptors, were used to amplify the V3-V4 region of the *16S rRNA* gene. MiSeq library preparation and sequencing were performed using a 2 × 300 cartridge (Illumina, Eindhoven, the Netherlands). A detailed description of the PCR protocol, DNA clean-up and library preparation is provided in Supplementary material 3. Sequencing data from Illumina software were processed by PANDAseq (version 2.5) and QIIME (version 1.7.0). ARB software (version 5.5) was used to detect contamination and the number of readouts. Samples were excluded in case the proportion of contamination exceeded 10% or if the number of reads was lower than 20,000. Alpha diversity (Shannon index) was calculated for taxonomic level of species, after total sum scaling (TSS). Prior to the taxon-wise association analysis, the data was transformed using centered log ratio transformation for each taxonomic level separately.

### Statistical Evaluation

Continuous variables are expressed as medians [IQR] and are compared with the Mann-Whitney *U*-test, unless stated otherwise. Categorical data were compared with chi-square or Fisher exact test, as appropriate. Relative abundances of gut microbial taxa were calculated and compared between BA patients and controls pre-KPE using linear regression adjusted for sex. Associations of beta diversity (Bray-Curtis dissimilarity index calculated using package ‘vegan’ v.2.5.6) and clinical outcome parameters was calculated using adonis (permutational multivariate analysis of variance using distance matrices), for each time point separately. To predict COJ with alpha diversity, previously mentioned clinical and biochemical parameters were analyzed by generalized linear models with logit link function (logistic regression), treating COJ as a binary outcome (COJ [+], COJ [−]). We used a forward stepwise approach using Akaike information criterion (AIC) values. The included clinical parameters were transformed using inverse rank transformation.

A 2-sided nominal *P* value <0.05 was considered statistically significant. Analyses were performed using R v.3.5.1 and IBM SPSS Statistics 23.0 (Armonk, NY).

## RESULTS

We included 6 (2 boys, 4 girls) patients who cleared their jaundice (COJ [+]) post-KPE. To these patients, we matched 6 (1 boy, 5 girls) patients who did not clear their jaundice (COJ[−]), according to age at KPE and gestational age (GA) (age at KPE, COJ[+] 1.5 [0.9–2.1] months vs COJ[−] 1.6 [1.3–1.8] months; *P* = 0.70, GA COJ[+] 39.0 [38.5–39.3] weeks vs 39.5 [38.9–40.0] weeks, *P* = 0.18). We selected age-matched control patients (6 boys; age at surgery 2.0 [1.4–2.1] months vs age at KPE 1.6 [1.3–1.8] in all BA patients *P* = 0.10). The mode of delivery was comparable between BA and controls (%vaginal delivery: BA 83%, controls 67%, *P* = 0.57). The proportion of patients born in urban or rural areas did not differ between BA and controls (6 [50%] of BA patients born in rural areas versus 2 [33%] in controls, *P* = 0.64), nor between COJ[−] and COJ[+] patients (3 [50%] vs 3 [50%], *P* = 1.00). Feeding method was comparable between BA and controls (2 [17%] breastfed BA patients vs 2 [33%] in controls, *P* = 0.57). Baseline parameters of BA and control patients are depicted in Supplementary material 4.

Baseline and follow-up parameters of COJ[+] and COJ[−] patients are depicted in Table 1. All parameters were statistically comparable, yet a trend towards lower AST levels in COJ[+] (168 [111–202]) versus COJ[−] (247 [166–379]; *P* = 0.08) was observed. LPS levels were a 10-fold higher in COJ[−], compared with COJ[+] (20.1 [2.6–458.0] vs 2.4 [1.8–60.4] mEU/mL, respectively; *P* = 0.27). Correspondingly, LBP (which binds LPS) levels were lower in COJ[−] patients (52 [15–192] vs 144 [26–953] ug/mL, respectively, *P* = 0.47). Both differences were not statistically significant.

**TABLE 1:** Baseline and follow-up characteristics in all biliary atresia patients before Kasai portoenterostomy

	COJ+ (n = 6)	COJ− (n = 6)	*P*
Males, n (%)	2 (33)	3 (50)	0.56
Gestational age, week	39.0 [38.4–39.2]	39.5 [38.9–40.0]	0.18
Diet			0.12
Exclusively breast milk, n (%)	0 (0)	2 (33)	
Formula, n (%)	6 (100)	4 (67)	
Urbanicity			1.00
Rural, n (%)	3 (50)	3 (50)	
Urban, n (%)	3 (50)	3 (50)	
Delivery method			0.46
Vaginal, n (%)	4 (67)	6 (100)	
C-section, n (%)	2 (33)	0 (0)	
Biochemistry at baseline			
TSB, μmol/L	143 [96–202]	138 [134–193]	0.87
ALT, U/L	128 [77–179]	177 [104–311]	0.26
AST, U/L	168 [111–202]	247 [166–379]	0.08
AP, U/L	594 [373–643]	566 [452–580]	0.72
GGT, U/L	407 [293–954]	280 [219–515]	0.27
Albumin, g/L	37 [36–40]	37 [36–38]	0.89
Platelet count, 10^9^/L	451 [248–531]	509 [422–638]	0.27
INR	[1.0–1.1]	0.9 [0.9–1.0]	0.06
APRi	0.74 [0.57–2.07]	1.23 [0.79–1.73]	0.72
LPS, mEU/mL	2.4 [1.8–60.4]	20.1 [2.6–458.0]	0.27
LBP, ug/mL	144 [26–953]	52 [15–192]	0.47
sCD14, ug/mL	2.1 [1.6–4.5]	1.8 [1.2–4.6]	0.58
Claudin-3, ng/mL	38 [31–44]	36 [28–42]	0.72
IFABP, pg/mL	2069 [1915–4542]	3562 [1619–6674]	0.47
IL-6, pg/mL	15 [0–683]	4 [0–14]	0.58
Age at KPE, months	1.5 [0.9–2.1]	1.6 [1.3–1.8]	0.75

Variables expressed as medians and interquartile ranges. ALT = alanine aminotransferase; AP = alkaline phosphatase; ARPi = AST to platelet ratio index; AST = aspartate aminotransferase; GGT = gamma glutamyl transpeptidase; IFABP = intestinal fatty acid binding protein; IL-6 = interleukin 6; INR = international normalized ratio; KPE = Kasai portoenterostomy; LBP = lipopolysaccharide binding protein; LPS = lipopolysaccharide; NLS = native liver survival; sCD14 = soluble cluster of differentiation 14.

At last known follow-up (age 16.7 [8.3–32.1] months), 7 patients (58%) had undergone LTx (of which 5 COJ[−] patients). As expected, COJ was associated with native liver survival (NLS) (%NLS at age 1 year: COJ[+] 100% vs COJ[−] 33%, *P* = 0.01).

### Comparable Alpha Diversity Yet Different Structure of Fecal Microbiota in Biliary Atresia Patients Versus Controls

**FIGURE 1:**
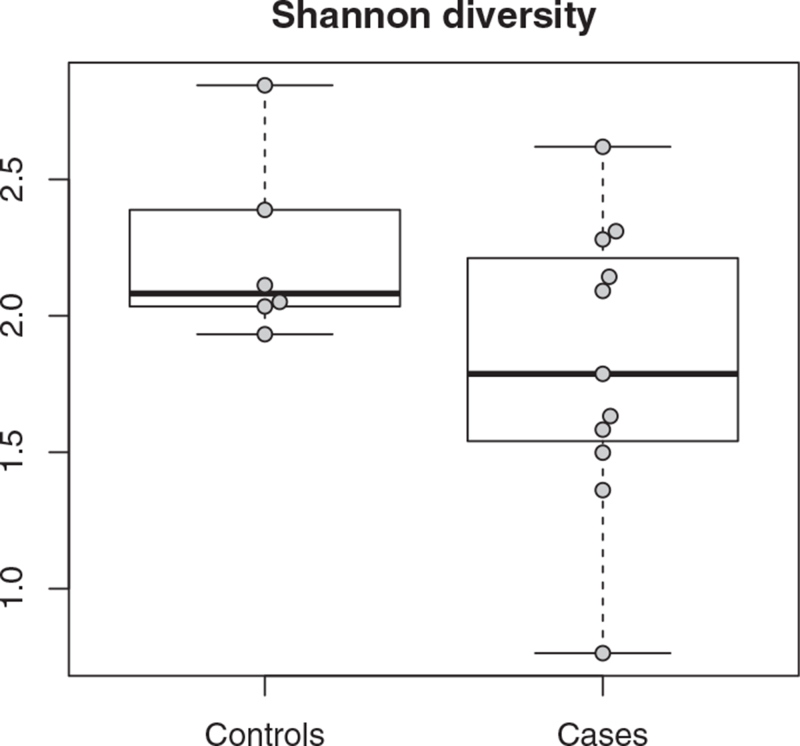
Alpha diversity on genus level of gut microbiota in controls (left, controls) and patients with biliary atresia (right, cases), before surgery (*P* = 0.34).

The alpha diversity of the microbiota of BA patients was comparable to that of controls (*P* = 0.22, Fig. 1). The taxonomic composition of the microbiota, however, differed. BA patients had lower abundances of, for example, families Bifidobacteriaceae (β = −1.98, *P* < 0.001) and Lachnospiraceae (β = −1.84, *P* = 0.007) and higher abundances of genus *Streptococcus* (β = 1.13, *P* = 0.003) compared with controls (Supplementary material 5).

### The Alpha Diversity and the Composition of the Fecal Microbiota Before Kasai are Associated With Clearance of Jaundice After Kasai

There were no significant differences in biochemistry pre-KPE between patients with and without COJ (Table 1). COJ[+] patients all received formula feeding before admission, while 2 of the 6 COJ [−] patients received breast-feeding only, and 4 received formula feeding (COJ[+] vs COJ[−] *P* = 0.12).

**FIGURE 2:**
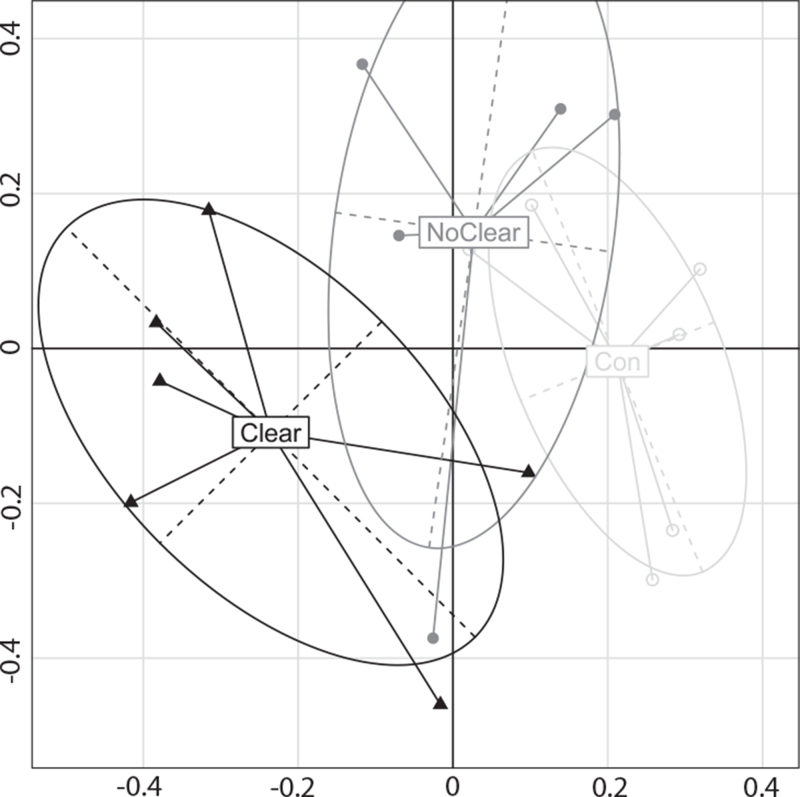
Principal coordinate analysis of microbial communities in patients with (‘Clear’, filled triangles) or without (‘NoClear’, filled circles) clearance of jaundice during follow-up, and controls (Con, open circles) at time point 1 (at presentation).

**FIGURE 3:**
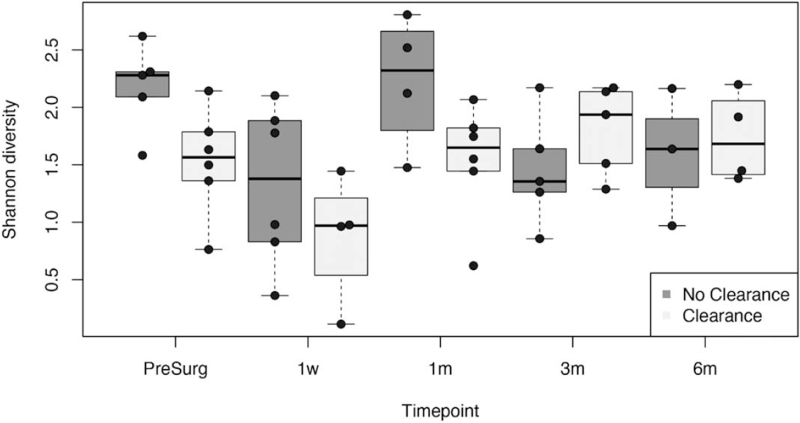
Shannon diversity of patients without clearance of jaundice (left columns) and with clearance of jaundice (right columns) during follow-up. PS *=* pre-surgery.

Figure 2 depicts a principal coordinate analysis plot of microbial communities of patients with and without COJ (and controls). We observed a lower alpha diversity pre-KPE in COJ[+] compared with COJ[−] patients (Fig. 3). Lower alpha diversity pre-KPE was associated with higher chance of clearance, COJ[+] (βlogit = −0.64, *P* = 0.04). In order to ascertain that alpha diversity may indeed be associated with COJ, we constructed a model to predict COJ based on pre-KPE parameters. We included clinical parameters (biochemistry at baseline, patient demographic data). Only alpha diversity and IFABP levels were selected (based on a forward stepwise approach using Akaike information criterion values) as the parameters that significantly contributed to prediction of COJ (Supplementary material 6).

In COJ[+] patients, we observed greater abundances of genus *Acinetobacter* (β = 1.27, *P* = 0.03) and family Clostridiaceae (β = 1.45, *P* = 0.03) and lower abundances of family *Enterobacteriaceae*, including genera *Klebsiella* [β = −1.21, *P* = 0.01], *Salmonella* (β = −1.57, *P* = 0.02) and *Trabulsiella* (β = −1.29, *P* = 0.006) (Supplementary Material 7). COJ[+] patients harbored lower abundances of *Bifidobacterium* (β = −1.44, *P* = 0.02).

### The Fecal Microbiota During Follow-up After Kasai Portoenterostomy

At 1 week post-KPE, alpha diversity decreased both in COJ[+] and COJ[−] patients compared with the sample obtained before KPE (Fig. 3). Alpha diversity between COJ[+] and [−] patients was comparable (*r* = −0.36, *P* = 0.31).

At 1 month post-KPE, alpha diversity had increased in both patient groups compared with 1 week post-KPE, despite the use of antibiotic prophylaxis in all patients. A trend towards lower alpha diversity was observed in COJ[+] compared with COJ[−] (*r* −0.57, *P* = 0.09, Fig. 3). Significantly lower abundance of *Bifidobacterium* was observed in the COJ [+] group (β = −1.82, *P* = 0.0009).

At 3 months post-KPE, alpha diversity decreased in COJ[−] and increased in COJ[+], compared with 1 month post-KPE. Alpha diversity in COJ[+] patients was comparable to that of COJ [−] patients (*r* = 0.31, *P* = 0.38, Fig. 3), yet increased abundances of *Firmicutes* (eg, Veillonellaceae and Clostridiaceae) were observed. At 6 months post-KPE, alpha diversity had not significantly changed in COJ[+], yet it had increased in COJ[−], compared with 3 months post-KPE (*r* = 0.14, *P* = 0.76).

## DISCUSSION

The role of the GM has become more closely implicated in the enterohepatic circulation, the gut-liver axis and in the progression of various liver diseases (4,5). We hypothesized that this may also apply to BA, and that there might be relationship between the GM composition before KPE and the COJ after KPE. This prospective pilot study demonstrates that the structure of the GM differs significantly between BA patients and controls. In addition, our results suggest that before KPE, the GM of BA patients contains predictive information on the chances of success of the KPE with respect to COJ.

In this study, the alpha diversity between BA patients and controls was comparable. Wang et al (11), who included 34 BA patients and 34 controls, have previously showed that alpha diversity in BA patients was significantly lower compared with that of controls. It might be that our sample size was too small to detect such significant differences.

The composition of the microbiota differed significantly: BA was associated with lower abundances of *Actinobacteria* (eg, Bifidobacteriaceae), the family Lachnospiraceae and greater abundances of Firmicutes (eg, Streptococcaceae), 3 families, which had been associated with BA previously (11). Bifidobacteriaceae, such as *Bifidobacterium longum and Bifidobacterium adolescentis,* are abundant anaerobic bacteria in the microbiota of healthy infants (15,16). These bacteria degrade complex polysaccharides to intermediate products of short-chain fatty acids, such as acetate and lactate (16). Family Lachnospiraceae produce butyrate, which contributes to gut barrier stability (16,17). A decrease in these bacteria could, therefore, increase translocation of bacterial products towards the liver. It might be that in BA such a mechanism is present, which may be partly responsible for the development of cirrhosis, even after successful KPE.

Apart from differences in GM between BA patients and controls, the present study found an intriguing association between the GM and outcome of the KPE. Already before KPE, the GM of patients who achieve COJ[+] was associated with a difference in alpha diversity and GM composition, compared with COJ[−] patients. The striking finding that the GM before KPE is associated with outcome after KPE has recently been suggested by work from Tessier et al (10). Specific bacteria have the ability to deconjugate bile and indirectly contribute to gut wall stability. Our data provide some support that increased gut wall permeability may be present in BA. The multivariate model, including clinical parameters pre-KPE, suggested that not only alpha diversity but also serum levels of IFABP before KPE have the most predictive power for forecasting COJ after KPE compared with other clinical pre-KPE parameters. IFABP levels were 2-fold higher in COJ[−] versus COJ[+], which may indicate increased enterocyte damage and potentially, increased bacterial translocation. Correspondingly, LPS levels were a 10-fold higher in COJ[−] patients. Despite the limitations of this study, we hypothesize that not only bile acid metabolism but also gut wall permeability may play a role in the success of KPE surgery and thus in the prognosis of BA. Our data indicate that future studies should include analysis of gut permeability and translocation of microbes or microbial products in BA, for example, by focusing on tight-junction protein in intestinal specimens, the presence of bacterial or viral genomic products in liver and its adjacent or mesenteric lymph nodes and intestinal bile acid composition, or a disruption therein (18).

Patients with COJ after KPE had significantly lower overall alpha diversity. This result is conflicting to so far published studies. Case series by Elaine Chen et al (10) and Tessier et al (12) report associations between achieving COJ and higher alpha diversity (12). Although decreased alpha diversity is generally considered unhealthy during the development of the GM (15), increased alpha diversity in BA, especially when predominated by dysbiotic microbiota, may not associate with improved outcome per se. The course of COJ[+] patients followed that of a healthy development of the infant GM (19), which may have followed the successful re-establishment of bile flow in the first 6 months.

In addition, patients with COJ harbored lower abundances of Enterobacteriaceae. Such bacteria have been associated with risk for cholangitis or sepsis in BA. A lack therein may, therefore, be beneficial for long-term outcome, which has been suggested in a recent and elegant review by Jain et al (20). These authors, among others, also associated *Bifidobacterium* with achieving COJ (10–12,20), which is to be expected because of their previously mentioned beneficial effects on gut wall stability. In our study, COJ[+] surprisingly associated with a decreased abundance of these bacteria. Although we do not have a conclusive explanation for this finding, we speculate that feeding method before presentation in our center (COJ[+]: all formula; COJ[−]: formula n = 4, breast milk n = 2) may have influenced these results.

The present study was limited by several factors. The rarity of BA, the single-center (although nationwide) and prospective character of this study resulted in a limited sample size. Furthermore, although no difference in urbanicity was observed between BA and controls, matching on urbanicity should be performed in future studies as the development of the microbiome is related to population density (19). We are aware that the present results are to be interpreted with caution. We nevertheless feel that our results provide worthwhile indications to target future studies. The strength of the present cohort consists of age-matched, term-born BA patients with the same anatomic variant (type 3), thereby minimizing patient variation as much as possible. Moreover, the Netherlands is a Western country in which large socioeconomic differences are absent. We, therefore, assume that feeding is rather comparable and adequate in this cohort.

In summary, our study demonstrates that, although alpha diversity was comparable, the structure of the GM of patients with BA differed from that of controls. Strikingly, the alpha diversity and structure of the GM differed already before KPE between patients with and without COJ after KPE. Our data are in agreement with the concept the GM composition, possibly via pathological bacterial translocation, is an important factor in defining the success or failure of KPE surgery with respect to COJ.

## Supplementary Material

Supplemental Digital Content
