# Supplementary material for: Gut Microbiota Composition of Biliary Atresia Patients Before Kasai Portoenterostomy Associates With Long-term Outcome
Source: J Pediatr Gastroenterol Nutr. 2021 Jul 14;73(4):485–90. doi: 10.1097/MPG.0000000000003234 (PMC8448407; doi:10.1097/MPG.0000000000003234)
Supplement: Supplemental Digital Content [file jpga-73-485-s001.docx]

**Supplementary material 1 - Enzyme-linked immunosorbent assay for intestinal integrity and translocation markers***LPS* was determined according to a chromogenic method in which endotoxin catalyzes the activation of a proenzyme in Limulus Amebocyte Lysate (LAL) (Genscript Piscataway, NJ, USA). A microtiterplate method required 10 μl samples and a standard curve starting at 0,75 EU/ml which is 1:1 diluted. The lowest standard was 0,01 EU/ml. Samples and standards were incubated for 30 minutes with LAL and then mixed with chromogenic substrate and color stabilizers. Conversion of substrate was read at 545 nm.

*LBP* was determined by means of sandwich ELISA based on LPS as capture and mouse anti LBP monoclonal antibody for detection (LSBio, Seattle, Wa, USA). The LPS, LBP, antibody complex was determined by HRP-labelled goat-anti mouse antibody (DAKO, Glostrup, Denmark) and ODP substrate. The standard curve had a highest concentration of 1000ng/ml. Samples were diluted 1:20 in 0,1% BSA/PBS buffer.

*IFABP* was determined by means of sandwich ELISA based on capture and biotin-labelled detection antibodies from R&D systems (Abingdon, UK). Streptavidin-HRP and OPD substrate was used to quantify the amount of IFABP. The standard curve had a highest concentration of 2 ng/ml. Samples were diluted 1:1 in 0,1% BSA/PBS buffer.

*sCD14* was determined by means of sandwich ELISA based on capture and biotin-labelled detection antibodies from R&D systems (Abingdon, UK). Streptavidin-HRP and OPD substrate was used to quantify the amount of CD14. The standard curve had a highest concentration of 4 ng/ml. Samples were diluted 1:2500 in 0,1% BSA/PBS buffer.

*Claudin-3* was performed by an ELISA test kit from Cloud-Clone Corp (Houston, TX, USA). The standard curve of claudine-3 had a highest concentration of 20 ng/ml. Samples were diluted 1:1 in assay buffer. TMB substrate was used to quantify the amount of Claudin-3.

*IL-6* was determined by means of sandwich ELISA based on capture and biotin-labelled detection antibodies from Biolegend (San Diego, Ca, USA). The standard curve of IL-6 had a highest concentration of 1000 pg/ml. Samples were diluted 1:1 in assay buffer. Streptavidin-poly HRP and ODP substrate were used to quantify the interleukin amount.

**Supplementary material 2 – Primers**

| V3_F_modified | aatgatacggcgaccaccgagatct |  | acactctttccctacacgacgctcttccgatct | | |  |  | NNNNCCTACGGGAGGCAGCAG |
| --- | --- | --- | --- | --- | --- | --- | --- | --- |
|  |  |  |  |  |  | |  |  |
| V4_1R | caagcagaagacggcatacgagat |  | **ATCACG** |  | gtgactggagttcagacgtgtgctcttccgatct | |  | GGACTACHVGGGTWTCTAAT |
|  |  |  |  |  |  | |  |  |
| V4_2R | caagcagaagacggcatacgagat |  | **CGATGT** |  | gtgactggagttcagacgtgtgctcttccgatct | |  | GGACTACHVGGGTWTCTAAT |
|  |  |  |  |  |  | |  |  |
| V4_3R | caagcagaagacggcatacgagat |  | **TTAGGC** |  | gtgactggagttcagacgtgtgctcttccgatct | |  | GGACTACHVGGGTWTCTAAT |
|  |  |  |  |  |  | |  |  |
| V4_4R | caagcagaagacggcatacgagat |  | **TGACCA** |  | gtgactggagttcagacgtgtgctcttccgatct | |  | GGACTACHVGGGTWTCTAAT |
|  |  |  |  |  |  | |  |  |
| V4_5R | caagcagaagacggcatacgagat |  | **ACAGTG** |  | gtgactggagttcagacgtgtgctcttccgatct | |  | GGACTACHVGGGTWTCTAAT |
|  |  |  |  |  |  | |  |  |
| V4_6R | caagcagaagacggcatacgagat |  | **GCCAAT** |  | gtgactggagttcagacgtgtgctcttccgatct | |  | GGACTACHVGGGTWTCTAAT |
|  |  |  |  |  |  | |  |  |
| V4_7R | caagcagaagacggcatacgagat |  | **CAGATC** |  | gtgactggagttcagacgtgtgctcttccgatct | |  | GGACTACHVGGGTWTCTAAT |
|  |  |  |  |  |  | |  |  |
| V4_8R | caagcagaagacggcatacgagat |  | **ACTTGA** |  | gtgactggagttcagacgtgtgctcttccgatct | |  | GGACTACHVGGGTWTCTAAT |
|  |  |  |  |  |  | |  |  |
| V4_9R | caagcagaagacggcatacgagat |  | **GATCAG** |  | gtgactggagttcagacgtgtgctcttccgatct | |  | GGACTACHVGGGTWTCTAAT |
|  |  |  |  |  |  | |  |  |
| V4_10R | caagcagaagacggcatacgagat |  | **TAGCTT** |  | gtgactggagttcagacgtgtgctcttccgatct | |  | GGACTACHVGGGTWTCTAAT |
|  |  |  |  |  |  | |  |  |
| V4_11R | caagcagaagacggcatacgagat |  | **GGCTAC** |  | gtgactggagttcagacgtgtgctcttccgatct | |  | GGACTACHVGGGTWTCTAAT |
|  |  |  |  |  |  | |  |  |
| V4_12R | caagcagaagacggcatacgagat |  | **CTTGTA** |  | gtgactggagttcagacgtgtgctcttccgatct | |  | GGACTACHVGGGTWTCTAAT |
|  |  |  |  |  |  | |  |  |
| V4_13R | caagcagaagacggcatacgagat |  | **AGTACG** |  | gtgactggagttcagacgtgtgctcttccgatct | |  | GGACTACHVGGGTWTCTAAT |
|  |  |  |  |  |  | |  |  |
| V4_14R | caagcagaagacggcatacgagat |  | **TCAGTC** |  | gtgactggagttcagacgtgtgctcttccgatct | |  | GGACTACHVGGGTWTCTAAT |
|  |  |  |  |  |  | |  |  |
| V4_15R | caagcagaagacggcatacgagat |  | **TTGAGC** |  | gtgactggagttcagacgtgtgctcttccgatct | |  | GGACTACHVGGGTWTCTAAT |
|  |  |  |  |  |  | |  |  |
| V4_16R | caagcagaagacggcatacgagat |  | **AAGCGA** |  | gtgactggagttcagacgtgtgctcttccgatct | |  | GGACTACHVGGGTWTCTAAT |
|  |  |  |  |  |  | |  |  |
| V4_17R | caagcagaagacggcatacgagat |  | **TCCTCA** |  | gtgactggagttcagacgtgtgctcttccgatct | |  | GGACTACHVGGGTWTCTAAT |
|  |  |  |  |  |  | |  |  |
| V4_18R | caagcagaagacggcatacgagat |  | **GGTTGT** |  | gtgactggagttcagacgtgtgctcttccgatct | |  | GGACTACHVGGGTWTCTAAT |
|  |  |  |  |  |  | |  |  |
| V4_19R | caagcagaagacggcatacgagat |  | **TGAGGT** |  | gtgactggagttcagacgtgtgctcttccgatct | |  | GGACTACHVGGGTWTCTAAT |
|  |  |  |  |  |  | |  |  |
| V4_20R | caagcagaagacggcatacgagat |  | **TACCGT** |  | gtgactggagttcagacgtgtgctcttccgatct | |  | GGACTACHVGGGTWTCTAAT |
|  |  |  |  |  |  | |  |  |
| V4_21R | caagcagaagacggcatacgagat |  | **CCAACT** |  | gtgactggagttcagacgtgtgctcttccgatct | |  | GGACTACHVGGGTWTCTAAT |
|  |  |  |  |  |  | |  |  |
| V4_22R | caagcagaagacggcatacgagat |  | **AGAGAG** |  | gtgactggagttcagacgtgtgctcttccgatct | |  | GGACTACHVGGGTWTCTAAT |
|  |  |  |  |  |  | |  |  |
| V4_23R | caagcagaagacggcatacgagat |  | **CACTTG** |  | gtgactggagttcagacgtgtgctcttccgatct | |  | GGACTACHVGGGTWTCTAAT |
|  |  |  |  |  |  | |  |  |
| V4_24R | caagcagaagacggcatacgagat |  | **TCAAGG** |  | gtgactggagttcagacgtgtgctcttccgatct | |  | GGACTACHVGGGTWTCTAAT |
|  |  |  |  |  |  | |  |  |
| V4_25R | caagcagaagacggcatacgagat |  | **AGTGGT** |  | gtgactggagttcagacgtgtgctcttccgatct | |  | GGACTACHVGGGTWTCTAAT |
|  |  |  |  |  |  | |  |  |
| V4_26R | caagcagaagacggcatacgagat |  | **GACACT** |  | gtgactggagttcagacgtgtgctcttccgatct | |  | GGACTACHVGGGTWTCTAAT |
|  |  |  |  |  |  | |  |  |
| V4_27R | caagcagaagacggcatacgagat |  | **CCTTCT** |  | gtgactggagttcagacgtgtgctcttccgatct | |  | GGACTACHVGGGTWTCTAAT |
|  |  |  |  |  |  | |  |  |
| V4_28R | caagcagaagacggcatacgagat |  | **GGATAA** |  | gtgactggagttcagacgtgtgctcttccgatct | |  | GGACTACHVGGGTWTCTAAT |
|  |  |  |  |  |  | |  |  |
| V4_29R | caagcagaagacggcatacgagat |  | **CCTTAA** |  | gtgactggagttcagacgtgtgctcttccgatct | |  | GGACTACHVGGGTWTCTAAT |
|  |  |  |  |  |  | |  |  |
| V4_30R | caagcagaagacggcatacgagat |  | **CAAGAA** |  | gtgactggagttcagacgtgtgctcttccgatct | |  | GGACTACHVGGGTWTCTAAT |
|  |  |  |  |  |  | |  |  |
| V4_31R | caagcagaagacggcatacgagat |  | **GTTGAA** |  | gtgactggagttcagacgtgtgctcttccgatct | |  | GGACTACHVGGGTWTCTAAT |
|  |  |  |  |  |  | |  |  |
| V4_32R | caagcagaagacggcatacgagat |  | **TCACAA** |  | gtgactggagttcagacgtgtgctcttccgatct | |  | GGACTACHVGGGTWTCTAAT |
|  |  |  |  |  |  | |  |  |
| V4_33R | caagcagaagacggcatacgagat |  | **AGTCAA** |  | gtgactggagttcagacgtgtgctcttccgatct | |  | GGACTACHVGGGTWTCTAAT |
|  |  |  |  |  |  | |  |  |
| V4_34R | caagcagaagacggcatacgagat |  | **CGAATA** |  | gtgactggagttcagacgtgtgctcttccgatct | |  | GGACTACHVGGGTWTCTAAT |
|  |  |  |  |  |  | |  |  |
| V4_35R | caagcagaagacggcatacgagat |  | **GCTATA** |  | gtgactggagttcagacgtgtgctcttccgatct | |  | GGACTACHVGGGTWTCTAAT |
|  |  |  |  |  |  | |  |  |
| V4_36R | caagcagaagacggcatacgagat |  | **GAGTTA** |  | gtgactggagttcagacgtgtgctcttccgatct | |  | GGACTACHVGGGTWTCTAAT |
|  |  |  |  |  |  | |  |  |
| V4_37R | caagcagaagacggcatacgagat |  | **TTGGTA** |  | gtgactggagttcagacgtgtgctcttccgatct | |  | GGACTACHVGGGTWTCTAAT |
|  |  |  |  |  |  |  |  |  |

| V4_38R | caagcagaagacggcatacgagat | **AACGTA** |  | gtgactggagttcagacgtgtgctcttccgatct |  | GGACTACHVGGGTWTCTAAT |
| --- | --- | --- | --- | --- | --- | --- |
|  |  |  |  |  |  |  |
| V4_39R | caagcagaagacggcatacgagat | **GTACTA** |  | gtgactggagttcagacgtgtgctcttccgatct |  | GGACTACHVGGGTWTCTAAT |
|  |  |  |  |  |  |  |
| V4_40R | caagcagaagacggcatacgagat | **CATCTA** |  | gtgactggagttcagacgtgtgctcttccgatct |  | GGACTACHVGGGTWTCTAAT |
|  |  |  |  |  |  |  |
| V4_41R | caagcagaagacggcatacgagat | **TGTAGA** |  | gtgactggagttcagacgtgtgctcttccgatct |  | GGACTACHVGGGTWTCTAAT |
|  |  |  |  |  |  |  |
| V4_42R | caagcagaagacggcatacgagat | **ATCAGA** |  | gtgactggagttcagacgtgtgctcttccgatct |  | GGACTACHVGGGTWTCTAAT |
|  |  |  |  |  |  |  |
| V4_43R | caagcagaagacggcatacgagat | **ACATGA** |  | gtgactggagttcagacgtgtgctcttccgatct |  | GGACTACHVGGGTWTCTAAT |
|  |  |  |  |  |  |  |
| V4_44R | caagcagaagacggcatacgagat | **TAGACA** |  | gtgactggagttcagacgtgtgctcttccgatct |  | GGACTACHVGGGTWTCTAAT |
|  |  |  |  |  |  |  |
| V4_45R | caagcagaagacggcatacgagat | **GAGAAT** |  | gtgactggagttcagacgtgtgctcttccgatct |  | GGACTACHVGGGTWTCTAAT |
|  |  |  |  |  |  |  |
| V4_46R | caagcagaagacggcatacgagat | **CTCAAT** |  | gtgactggagttcagacgtgtgctcttccgatct |  | GGACTACHVGGGTWTCTAAT |
|  |  |  |  |  |  |  |
| V4_47R | caagcagaagacggcatacgagat | **AGGTAT** |  | gtgactggagttcagacgtgtgctcttccgatct |  | GGACTACHVGGGTWTCTAAT |
|  |  |  |  |  |  |  |
| V4_48R | caagcagaagacggcatacgagat | **TTGCAT** |  | gtgactggagttcagacgtgtgctcttccgatct |  | GGACTACHVGGGTWTCTAAT |
|  |  |  |  |  |  |  |
| V4_49R | caagcagaagacggcatacgagat | **TGGATT** |  | gtgactggagttcagacgtgtgctcttccgatct |  | GGACTACHVGGGTWTCTAAT |
|  |  |  |  |  |  |  |
| V4_50R | caagcagaagacggcatacgagat | **ACCATT** |  | gtgactggagttcagacgtgtgctcttccgatct |  | GGACTACHVGGGTWTCTAAT |
|  |  |  |  |  |  |  |
| V4_51R | caagcagaagacggcatacgagat | **CTAGTT** |  | gtgactggagttcagacgtgtgctcttccgatct |  | GGACTACHVGGGTWTCTAAT |
|  |  |  |  |  |  |  |
| V4_52R | caagcagaagacggcatacgagat | **AGTGTT** |  | gtgactggagttcagacgtgtgctcttccgatct |  | GGACTACHVGGGTWTCTAAT |
|  |  |  |  |  |  |  |
| V4_53R | caagcagaagacggcatacgagat | **TCTCTT** |  | gtgactggagttcagacgtgtgctcttccgatct |  | GGACTACHVGGGTWTCTAAT |
|  |  |  |  |  |  |  |
| V4_54R | caagcagaagacggcatacgagat | **GTAAGT** |  | gtgactggagttcagacgtgtgctcttccgatct |  | GGACTACHVGGGTWTCTAAT |
|  |  |  |  |  |  |  |
| V4_55R | caagcagaagacggcatacgagat | **CAATGT** |  | gtgactggagttcagacgtgtgctcttccgatct |  | GGACTACHVGGGTWTCTAAT |
|  |  |  |  |  |  |  |
| V4_56R | caagcagaagacggcatacgagat | **ATTCGT** |  | gtgactggagttcagacgtgtgctcttccgatct |  | GGACTACHVGGGTWTCTAAT |
|  |  |  |  |  |  |  |
| V4_57R | caagcagaagacggcatacgagat | **ATGACT** |  | gtgactggagttcagacgtgtgctcttccgatct |  | GGACTACHVGGGTWTCTAAT |
|  |  |  |  |  |  |  |
| V4_58R | caagcagaagacggcatacgagat | **ACTTCT** |  | gtgactggagttcagacgtgtgctcttccgatct |  | GGACTACHVGGGTWTCTAAT |
|  |  |  |  |  |  |  |
| V4_59R | caagcagaagacggcatacgagat | **CATAAG** |  | gtgactggagttcagacgtgtgctcttccgatct |  | GGACTACHVGGGTWTCTAAT |
|  |  |  |  |  |  |  |
| V4_60R | caagcagaagacggcatacgagat | **TTCTAG** |  | gtgactggagttcagacgtgtgctcttccgatct |  | GGACTACHVGGGTWTCTAAT |
|  |  |  |  |  |  |  |
| V4_61R | caagcagaagacggcatacgagat | **AAGATG** |  | gtgactggagttcagacgtgtgctcttccgatct |  | GGACTACHVGGGTWTCTAAT |
|  |  |  |  |  |  |  |
| V4_62R | caagcagaagacggcatacgagat | **TATGTG** |  | gtgactggagttcagacgtgtgctcttccgatct |  | GGACTACHVGGGTWTCTAAT |
|  |  |  |  |  |  |  |
| V4_63R | caagcagaagacggcatacgagat | **AATTGG** |  | gtgactggagttcagacgtgtgctcttccgatct |  | GGACTACHVGGGTWTCTAAT |
|  |  |  |  |  |  |  |
| V4_64R | caagcagaagacggcatacgagat | **TAATCG** |  | gtgactggagttcagacgtgtgctcttccgatct |  | GGACTACHVGGGTWTCTAAT |
|  |  |  |  |  |  |  |
| V4_65R | caagcagaagacggcatacgagat | **ACTAAC** |  | gtgactggagttcagacgtgtgctcttccgatct |  | GGACTACHVGGGTWTCTAAT |
|  |  |  |  |  |  |  |
| V4_66R | caagcagaagacggcatacgagat | **TGTTAC** |  | gtgactggagttcagacgtgtgctcttccgatct |  | GGACTACHVGGGTWTCTAAT |
|  |  |  |  |  |  |  |
| V4_67R | caagcagaagacggcatacgagat | **ATACAC** |  | gtgactggagttcagacgtgtgctcttccgatct |  | GGACTACHVGGGTWTCTAAT |
|  |  |  |  |  |  |  |
| V4_68R | caagcagaagacggcatacgagat | **CTTATC** |  | gtgactggagttcagacgtgtgctcttccgatct |  | GGACTACHVGGGTWTCTAAT |
|  |  |  |  |  |  |  |
| V4_69R | caagcagaagacggcatacgagat | **AGATTC** |  | gtgactggagttcagacgtgtgctcttccgatct |  | GGACTACHVGGGTWTCTAAT |
|  |  |  |  |  |  |  |
| V4_70R | caagcagaagacggcatacgagat | **ACGGAA** |  | gtgactggagttcagacgtgtgctcttccgatct |  | GGACTACHVGGGTWTCTAAT |
|  |  |  |  |  |  |  |
| V4_71R | caagcagaagacggcatacgagat | **TGCGAA** |  | gtgactggagttcagacgtgtgctcttccgatct |  | GGACTACHVGGGTWTCTAAT |
|  |  |  |  |  |  |  |
| V4_72R | caagcagaagacggcatacgagat | **GACCAA** |  | gtgactggagttcagacgtgtgctcttccgatct |  | GGACTACHVGGGTWTCTAAT |
|  |  |  |  |  |  |  |
| V4_73R | caagcagaagacggcatacgagat | **CTGTCA** |  | gtgactggagttcagacgtgtgctcttccgatct |  | GGACTACHVGGGTWTCTAAT |
|  |  |  |  |  |  |  |
| V4_74R | caagcagaagacggcatacgagat | **GCAGAT** |  | gtgactggagttcagacgtgtgctcttccgatct |  | GGACTACHVGGGTWTCTAAT |
|  |  |  |  |  |  |  |
| V4_75R | caagcagaagacggcatacgagat | **TCGTGT** |  | gtgactggagttcagacgtgtgctcttccgatct |  | GGACTACHVGGGTWTCTAAT |
|  |  |  |  |  |  |  |
| V4_76R | caagcagaagacggcatacgagat | **GAACCT** |  | gtgactggagttcagacgtgtgctcttccgatct |  | GGACTACHVGGGTWTCTAAT |
|  |  |  |  |  |  |  |

| V4_77R | caagcagaagacggcatacgagat | **GTCATG** |  | gtgactggagttcagacgtgtgctcttccgatct |  | GGACTACHVGGGTWTCTAAT |
| --- | --- | --- | --- | --- | --- | --- |
|  |  |  |  |  |  |  |
| V4_78R | caagcagaagacggcatacgagat | **GATAGC** |  | gtgactggagttcagacgtgtgctcttccgatct |  | GGACTACHVGGGTWTCTAAT |
|  |  |  |  |  |  |  |
| V4_79R | caagcagaagacggcatacgagat | **AAGTCC** |  | gtgactggagttcagacgtgtgctcttccgatct |  | GGACTACHVGGGTWTCTAAT |
|  |  |  |  |  |  |  |
| V4_80R | caagcagaagacggcatacgagat | **ATTGCC** |  | gtgactggagttcagacgtgtgctcttccgatct |  | GGACTACHVGGGTWTCTAAT |
|  |  |  |  |  |  |  |
| V4_81R | caagcagaagacggcatacgagat | **CCGAGA** |  | gtgactggagttcagacgtgtgctcttccgatct |  | GGACTACHVGGGTWTCTAAT |
|  |  |  |  |  |  |  |
| V4_82R | caagcagaagacggcatacgagat | **CGCTGA** |  | gtgactggagttcagacgtgtgctcttccgatct |  | GGACTACHVGGGTWTCTAAT |
|  |  |  |  |  |  |  |
| V4_83R | caagcagaagacggcatacgagat | **GGCACA** |  | gtgactggagttcagacgtgtgctcttccgatct |  | GGACTACHVGGGTWTCTAAT |
|  |  |  |  |  |  |  |
| V4_84R | caagcagaagacggcatacgagat | **CGTGCA** |  | gtgactggagttcagacgtgtgctcttccgatct |  | GGACTACHVGGGTWTCTAAT |
|  |  |  |  |  |  |  |
| V4_85R | caagcagaagacggcatacgagat | **GGCCTT** |  | gtgactggagttcagacgtgtgctcttccgatct |  | GGACTACHVGGGTWTCTAAT |
|  |  |  |  |  |  |  |
| V4_86R | caagcagaagacggcatacgagat | **CCTGGT** |  | gtgactggagttcagacgtgtgctcttccgatct |  | GGACTACHVGGGTWTCTAAT |
|  |  |  |  |  |  |  |
| V4_87R | caagcagaagacggcatacgagat | **CAGGCT** |  | gtgactggagttcagacgtgtgctcttccgatct |  | GGACTACHVGGGTWTCTAAT |
|  |  |  |  |  |  |  |
| V4_88R | caagcagaagacggcatacgagat | **GTCGCT** |  | gtgactggagttcagacgtgtgctcttccgatct |  | GGACTACHVGGGTWTCTAAT |
|  |  |  |  |  |  |  |
| V4_89R | caagcagaagacggcatacgagat | **GCGTAG** |  | gtgactggagttcagacgtgtgctcttccgatct |  | GGACTACHVGGGTWTCTAAT |
|  |  |  |  |  |  |  |
| V4_90R | caagcagaagacggcatacgagat | **CTGGAG** |  | gtgactggagttcagacgtgtgctcttccgatct |  | GGACTACHVGGGTWTCTAAT |
|  |  |  |  |  |  |  |
| V4_91R | caagcagaagacggcatacgagat | **CTACGG** |  | gtgactggagttcagacgtgtgctcttccgatct |  | GGACTACHVGGGTWTCTAAT |
|  |  |  |  |  |  |  |
| V4_92R | caagcagaagacggcatacgagat | **ACACCG** |  | gtgactggagttcagacgtgtgctcttccgatct |  | GGACTACHVGGGTWTCTAAT |
|  |  |  |  |  |  |  |
| V4_93R | caagcagaagacggcatacgagat | **GTTCCG** |  | gtgactggagttcagacgtgtgctcttccgatct |  | GGACTACHVGGGTWTCTAAT |
|  |  |  |  |  |  |  |
| V4_94R | caagcagaagacggcatacgagat | **CAGCAC** |  | gtgactggagttcagacgtgtgctcttccgatct |  | GGACTACHVGGGTWTCTAAT |
|  |  |  |  |  |  |  |
| V4_95R | caagcagaagacggcatacgagat | **CCGTTC** |  | gtgactggagttcagacgtgtgctcttccgatct |  | GGACTACHVGGGTWTCTAAT |
|  |  |  |  |  |  |  |
| V4_96R | caagcagaagacggcatacgagat | **GCATCC** |  | gtgactggagttcagacgtgtgctcttccgatct |  | GGACTACHVGGGTWTCTAAT |
|  |  |  |  |  |  |  |
| V4_97R | caagcagaagacggcatacgagat | **TACGCC** |  | gtgactggagttcagacgtgtgctcttccgatct |  | GGACTACHVGGGTWTCTAAT |
|  |  |  |  |  |  |  |

*Lowercase letters denote adapter sequences necessary for binding to the flow cell, underlined lowercase are binding sites for the Illumina sequencing primers, bold uppercase highlight the index sequences (all the indexes were obtained from Illumina) and regular uppercase are the V3 region forward primer 341F and the V4 region reverse primers 806R. The inclusion of four maximally degenerated bases (“NNNN”) maximizes diversity during the first four bases of the run.*

**Supplementary material 3 - PCR protocol, the DNA clean-up and library preparation**

Reaction conditions consisted of an initial 94 °C for 3 min followed by 32 cycles of 94 °C for 45 sec, 50 °C for 60 sec, and 72 °C for 90 sec, and a final extension of 72 °C for 10 min. An agarose gel confirmed the presence of the product (band at ~465 base pairs) in successfully amplified samples. The remainder of the PCR product (~45 μl) of each sample was mixed thoroughly with 25 μl Agencourt AMPure XP magnetic beads (Beckman Coulter) and were incubated at room temperature for 5 minutes. Beads were subsequently separated from the solution by placing the tubes in a magnetic bead separator for 2 minutes. After discarding the cleared solution the beads were washed twice by suspending them in 200 μl freshly prepared 80% ethanol, incubating the tubes for 30s in the magnetic bead separator and subsequently discarding the cleared solution. The pellet was then air dried for 15 minutes and suspended in 52.5 μl 10 mM Tris HCl pH 8.5 buffer. Fifty μl of the cleared up solution are subsequently transferred to a new tube. The DNA concentration of each sample was done using a Qubit® 2.0 fluorometer (www.invitrogen.com/qubit) and the remainder of the sample was stored at -20 °C until library normalization. Library normalization was done the day before running samples on the MiSeq by making 2 nM dilutions of each sample. Samples were pooled together by combining 5 μl of each diluted sample. Ten μl of the pooled samples and 10 μl 0.2 M NaOH were subsequently combined and incubated for 5 minutes to denature the sample DNA. To this, 980 μl of the HT1 buffer from the MiSeq 2x300 kit is was subsequently added. A denatured diluted PhiX solution was made by combining 2 μl of a 10 nM PhiX library with 3 μl 10 mM Tris HCl pH 8.5 buffer with 0.1% Tween 20. These 5 μl were mixed with 5 μl 0.2 M NaOH and incubated for 5 minutes at room temperature. These 10 μl were subsequently mixed with 990 μl HT1 buffer. From the diluted sample pool, 150 μl were combined with 50 μl of the diluted PhiX solution and further diluted by adding 800 μl HT1 buffer. Finally, 600 μl of the prepared library were loaded into the sample loading reservoir of the MiSeq 2x300 cartridge.

|  | **BA  (n=12)** | **HC (n=6)** | ***P*** |
| --- | --- | --- | --- |
| **Males**, n (%) | 3 (33) | 6 (100) | 0.04 |
| **Gestational** **age**, wk | 39.1 [38.8-39.9] | 37.7 [37.6-38.2] | 0.002 |
| **Diet**   Exclusively breast milk, n (%)  Formula, n (%) | 2 (17)  10 (83) | 2 (33) 4 (67) | 0.57 |
| **Urbanicity**  Rural, n (%)  Urban, n (%) | 6 (50)  6 (50) | 2 (33)  4 (67) | 0.64 |
| **Delivery method**  Vaginal, n (%)  C-section, n (%) | 10 (83)  2 (17) | 4 (67)  2 (33) | 0.57 |
| **Age at surgery**, mo | 1.6 [1.3-1.8] | 2.0 [1.4-2.1] | 0.10 |

**Supplementary material 4.** Baseline and follow-up characteristics in all biliary atresia (BA) patients and healthy controls (HC). Continuous variables expressed as medians and interquartile ranges.

**Supplementary material 5 – Significantly differing abundances of samples of controls *versus* children with biliary atresia prior to Kasai portoenterostomy.**

| **Taxon** | **Beta** | **Standard error** | **F** | **P** | **Adjusted P** |
| --- | --- | --- | --- | --- | --- |
| k__Bacteria;p__Actinobacteria;c__Actinobacteria;o__Bifidobacteriales;f__Bifidobacteriaceae;Other;Other | -1.9826727 | 0.4089350 | -4.848380 | 0.0002580 | 0.1254971 |
| k__Bacteria;p__Actinobacteria;c__Actinobacteria;o__Bifidobacteriales;f__Bifidobacteriaceae;Other | -1.9541419 | 0.4133492 | -4.727581 | 0.0003239 | 0.1254971 |
| k__Bacteria;p__Firmicutes;c__Clostridia;o**Clostridiales;f**[Tissierellaceae];g__Finegoldia | 1.7839288 | 0.4140140 | 4.308861 | 0.0007211 | 0.1495364 |
| k__Bacteria;p__Firmicutes;c__Clostridia;o**Clostridiales;f**[Tissierellaceae];g**Finegoldia;s** | 1.7779548 | 0.4160186 | 4.273739 | 0.0007718 | 0.1495364 |
| k__Bacteria;p__Actinobacteria;c__Actinobacteria;o__Actinomycetales;f__Corynebacteriaceae;g**Corynebacterium;s** | 1.8798623 | 0.4752314 | 3.955678 | 0.0014357 | 0.2225268 |
| k__Bacteria;p__Actinobacteria;c__Actinobacteria;o__Actinomycetales;f__Corynebacteriaceae;g__Corynebacterium | 1.7638724 | 0.4721941 | 3.735482 | 0.0022161 | 0.2471675 |
| k__Bacteria;p__Actinobacteria;c__Actinobacteria;o__Bifidobacteriales;f__Bifidobacteriaceae;g__Bifidobacterium;s__adolescentis | -2.3340263 | 0.6254488 | -3.731762 | 0.0022325 | 0.2471675 |
| k__Bacteria;p__Actinobacteria;c__Actinobacteria;o__Actinomycetales;f__Corynebacteriaceae | 1.7676741 | 0.4845240 | 3.648269 | 0.0026339 | 0.2551570 |
| k__Bacteria;p__Firmicutes;c__Bacilli;o__Lactobacillales;f__Streptococcaceae;g**Streptococcus;s** | 1.1267201 | 0.3198648 | 3.522489 | 0.0033807 | 0.2894236 |
| k__Bacteria;p__Firmicutes;c__Clostridia;o**Clostridiales;f**[Tissierellaceae];g**Anaerococcus;s** | 1.4995628 | 0.4401160 | 3.407199 | 0.0042516 | 0.2894236 |
| k__Bacteria;p__Firmicutes;c__Clostridia;o**Clostridiales;f**[Tissierellaceae];g__Anaerococcus | 1.4931205 | 0.4464056 | 3.344762 | 0.0048139 | 0.2894236 |
| k__Bacteria;p__Firmicutes;c__Bacilli;o__Lactobacillales;f__Streptococcaceae | 1.0663487 | 0.3191867 | 3.340830 | 0.0048517 | 0.2894236 |
| k__Bacteria;p__Firmicutes;c__Bacilli;o__Lactobacillales;f__Streptococcaceae;g__Streptococcus | 1.0073264 | 0.3015494 | 3.340502 | 0.0048548 | 0.2894236 |
| k__Bacteria;p__Firmicutes;c__Clostridia;o__Clostridiales;f__Clostridiaceae;g__ | -1.8097749 | 0.5685624 | -3.183072 | 0.0066404 | 0.3248718 |
| k__Bacteria;p__Firmicutes;c__Clostridia;o**Clostridiales;f**[Tissierellaceae] | 1.4157505 | 0.4458643 | 3.175295 | 0.0067438 | 0.3248718 |
| k__Bacteria;Other | 0.9512876 | 0.3012070 | 3.158251 | 0.0069763 | 0.3248718 |
| k__Bacteria;p__Firmicutes;c__Clostridia;o__Clostridiales;f__Lachnospiraceae | -1.8402710 | 0.5846672 | -3.147553 | 0.0071262 | 0.3248718 |
| k__Bacteria;Other;Other;Other;Other;Other;Other | 0.8301314 | 0.2717902 | 3.054309 | 0.0085761 | 0.3635524 |
| k__Bacteria;p__Firmicutes;c__Clostridia;o__Clostridiales;f__Clostridiaceae;g**;s** | -1.7398577 | 0.5732837 | -3.034898 | 0.0089129 | 0.3635524 |
| k__Bacteria;p__Firmicutes;c__Bacilli;o__Lactobacillales;f__Streptococcaceae;g__Streptococcus;s__luteciae | -1.1082175 | 0.3734864 | -2.967223 | 0.0101923 | 0.3949534 |
| k__Bacteria;p__Firmicutes | 1.0944289 | 0.3752847 | 2.916263 | 0.0112738 | 0.4160552 |
| k__Bacteria;p__Firmicutes;c__Clostridia;o__Clostridiales;f__Lachnospiraceae;Other | -1.4960054 | 0.5249877 | -2.849601 | 0.0128600 | 0.4403982 |
| k__Bacteria;p__Proteobacteria;c__Betaproteobacteria;o__Neisseriales;f__Neisseriaceae;g**Eikenella;s** | 1.6449740 | 0.5789321 | 2.841394 | 0.0130699 | 0.4403982 |
| k__Bacteria;p__Firmicutes;c__Clostridia;o__Clostridiales;f__Lachnospiraceae;Other;Other | -1.4543194 | 0.5239822 | -2.775513 | 0.0148801 | 0.4624827 |
| k__Bacteria;p__Firmicutes;c__Clostridia;o__Clostridiales;f__Ruminococcaceae;g__Ruminococcus | -1.5683095 | 0.5685117 | -2.758623 | 0.0153823 | 0.4624827 |
| k__Bacteria;Other;Other;Other;Other;Other | 0.7886280 | 0.2868293 | 2.749468 | 0.0156613 | 0.4624827 |
| k__Bacteria;p__Firmicutes;c__Bacilli;o__Bacillales;Other;Other;Other | 0.9007870 | 0.3348788 | 2.689890 | 0.0176016 | 0.4624827 |
| k__Bacteria;p__Firmicutes;c__Clostridia;o__Clostridiales;f__Lachnospiraceae;g__[Ruminococcus];s__gnavus | -1.8803851 | 0.6994139 | -2.688516 | 0.0176490 | 0.4624827 |
| k__Bacteria;p__Firmicutes;c__Clostridia;o__Clostridiales;f__Lachnospiraceae;g__[Ruminococcus] | -1.8933549 | 0.7052147 | -2.684792 | 0.0177781 | 0.4624827 |
| k__Bacteria;p__Actinobacteria;c__Coriobacteriia;o__Coriobacteriales;f__Coriobacteriaceae;g__Atopobium;s__rimae | 2.1977871 | 0.8196949 | 2.681226 | 0.0179026 | 0.4624827 |
| k__Bacteria;p__Actinobacteria;c__Actinobacteria | -1.1451096 | 0.4482701 | -2.554508 | 0.0229161 | 0.5585501 |
| k__Bacteria;p__Firmicutes;c__Bacilli;o__Bacillales;Other;Other | 0.8845742 | 0.3467258 | 2.551221 | 0.0230627 | 0.5585501 |
| k__Bacteria;p__Bacteroidetes;c__Bacteroidia;o**Bacteroidales;f**[Paraprevotellaceae] | -1.7568541 | 0.6994936 | -2.511609 | 0.0249016 | 0.5713326 |
| k__Bacteria;p__Proteobacteria;c__Betaproteobacteria;o__Neisseriales;f__Neisseriaceae;g__Eikenella | 1.4649451 | 0.5840558 | 2.508228 | 0.0250649 | 0.5713326 |
| k__Bacteria;p__Firmicutes;Other;Other;Other;Other;Other | 1.0486543 | 0.4307242 | 2.434631 | 0.0288849 | 0.6213116 |
| k__Bacteria;p__Firmicutes;Other;Other;Other;Other | 1.0370851 | 0.4290478 | 2.417178 | 0.0298691 | 0.6213116 |
| k__Bacteria;p__Firmicutes;Other;Other;Other | 1.0378135 | 0.4362220 | 2.379095 | 0.0321287 | 0.6213116 |
| k__Bacteria;p__Firmicutes;c__Bacilli;o__Bacillales;Other | 0.8620788 | 0.3625592 | 2.377760 | 0.0322108 | 0.6213116 |
| k__Bacteria;p__Firmicutes;Other;Other | 1.0415150 | 0.4408958 | 2.362270 | 0.0331779 | 0.6213116 |
| k__Bacteria;p__Actinobacteria | -1.1053998 | 0.4682648 | -2.360630 | 0.0332819 | 0.6213116 |
| k__Bacteria;p__Firmicutes;c__Bacilli;o__Lactobacillales | 0.9387498 | 0.4004366 | 2.344316 | 0.0343333 | 0.6213116 |
| k__Bacteria;p__Firmicutes;c__Clostridia;o__Clostridiales;f__Veillonellaceae;g**Veillonella;s** | 1.2423526 | 0.5309428 | 2.339899 | 0.0346233 | 0.6213116 |
| k__Bacteria;p__Firmicutes;Other | 1.0618101 | 0.4562096 | 2.327461 | 0.0354525 | 0.6213116 |
| k__Bacteria;p__Proteobacteria;c__Gammaproteobacteria;o__Pasteurellales;f__Pasteurellaceae;Other | -1.2596249 | 0.5426558 | -2.321222 | 0.0358755 | 0.6213116 |
| k__Bacteria;p__Firmicutes;c__Clostridia;o__Clostridiales;f__Lachnospiraceae;g__Moryella | 1.4732236 | 0.6354794 | 2.318287 | 0.0360762 | 0.6213116 |
| k__Bacteria;p__Bacteroidetes;c__Bacteroidia;o**Bacteroidales;f**[Paraprevotellaceae];g__[Prevotella] | -1.7845849 | 0.7817520 | -2.282802 | 0.0385872 | 0.6477494 |
| k__Bacteria;p__Proteobacteria;c__Gammaproteobacteria;o__Pasteurellales;f__Pasteurellaceae;Other;Other | -1.2372516 | 0.5442403 | -2.273355 | 0.0392829 | 0.6477494 |
| k__Bacteria;Other;Other;Other;Other | 0.7389464 | 0.3309630 | 2.232716 | 0.0424125 | 0.6779277 |
| k__Bacteria;p__Actinobacteria;c__Coriobacteriia;o__Coriobacteriales;f__Coriobacteriaceae;g__Atopobium;Other | 2.1009983 | 0.9433761 | 2.227106 | 0.0428625 | 0.6779277 |
| k__Bacteria;Other;Other | 0.7663205 | 0.3540776 | 2.164273 | 0.0482168 | 0.6813402 |
| k__Bacteria;p__Firmicutes;c__Bacilli;o__Bacillales;f__Staphylococcaceae;g__Staphylococcus;s__haemolyticus | 0.9000849 | 0.4179180 | 2.153735 | 0.0491736 | 0.6813402 |
| k__Bacteria;p__Firmicutes;c__Clostridia;o**Clostridiales;f**[Tissierellaceae];g**Parvimonas;s** | 0.7318401 | 0.3398004 | 2.153735 | 0.0491736 | 0.6813402 |
| k__Bacteria;p__Actinobacteria;c__Actinobacteria;o__Actinomycetales;f__Corynebacteriaceae;g__Corynebacterium;s__aurimucosum | 1.4803561 | 0.6873435 | 2.153735 | 0.0491736 | 0.6813402 |

**Supplementary material 6 - summary statistics for each clinical phenotype predictor from the single predictor logistic regression models, and logistic regression effect sizes/P-values for the joint model.**

**
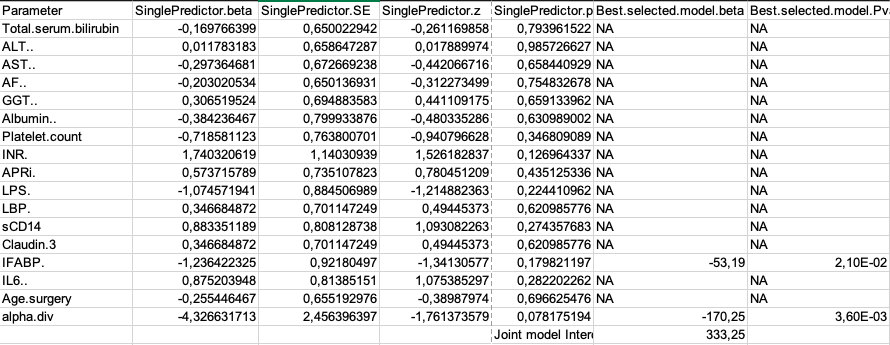
**

**Supplementary material 7 – Significantly differing abundances of samples of biliary atresia patient with clearance of jaundice *versus* patients without clearance of jaundice during follow-up, prior to Kasai portoenterostomy.**

| **Taxon** | **Beta** | **Standard error** | **F** | **P** | **Adjusted P** |
| --- | --- | --- | --- | --- | --- |
| k__Bacteria;p__Proteobacteria;c__Gammaproteobacteria;o__Enterobacteriales;f__Enterobacteriaceae;g__Trabulsiella | -1.174281 | 0.3005788 | -3.906732 | 0.0045017 | 0.8122951 |
| k__Bacteria;p__Proteobacteria;c__Gammaproteobacteria;o__Enterobacteriales;f__Enterobacteriaceae;g**Trabulsiella;s** | -1.287566 | 0.3463707 | -3.717307 | 0.0058941 | 0.8122951 |
| k__Bacteria;p__Actinobacteria;c__Coriobacteriia;o__Coriobacteriales;f__Coriobacteriaceae;Other | -1.691823 | 0.4850094 | -3.488227 | 0.0082198 | 0.8122951 |
| k__Bacteria;p__Actinobacteria;c__Coriobacteriia;o__Coriobacteriales;f__Coriobacteriaceae;Other;Other | -1.690506 | 0.5105312 | -3.311268 | 0.0106780 | 0.8122951 |
| k__Bacteria;p__Proteobacteria;c__Gammaproteobacteria;o__Enterobacteriales;f__Enterobacteriaceae;g__Klebsiella;Other | -1.216864 | 0.3859333 | -3.153042 | 0.0135362 | 0.8122951 |
| k__Bacteria;p__Actinobacteria;Other;Other | -1.651044 | 0.5318870 | -3.104125 | 0.0145745 | 0.8122951 |
| k__Bacteria;p__Actinobacteria;c__Actinobacteria;o__Bifidobacteriales;f__Bifidobacteriaceae;g**Bifidobacterium;s** | -1.441563 | 0.4685712 | -3.076509 | 0.0151971 | 0.8122951 |
| k__Bacteria;p__Actinobacteria;Other;Other;Other | -1.630770 | 0.5403958 | -3.017733 | 0.0166167 | 0.8122951 |
| k__Bacteria;p__Proteobacteria;c__Gammaproteobacteria;o__Enterobacteriales;f__Enterobacteriaceae;g**Salmonella;s** | -1.576804 | 0.5308980 | -2.970069 | 0.0178693 | 0.8122951 |
| k__Bacteria;p__Actinobacteria;Other | -1.543339 | 0.5310608 | -2.906144 | 0.0197052 | 0.8122951 |
| k__Bacteria;p__Actinobacteria;c__Actinobacteria;o__Bifidobacteriales;f__Bifidobacteriaceae;g__Bifidobacterium;Other | -1.307600 | 0.4586189 | -2.851168 | 0.0214405 | 0.8122951 |
| k__Bacteria;p__Actinobacteria;Other;Other;Other;Other | -1.605200 | 0.5728423 | -2.802166 | 0.0231205 | 0.8122951 |
| k__Bacteria;p__Proteobacteria;c__Gammaproteobacteria;o__Enterobacteriales;f__Enterobacteriaceae;g__Salmonella | -1.318828 | 0.4865106 | -2.710789 | 0.0266256 | 0.8122951 |
| k__Bacteria;p__Actinobacteria;Other;Other;Other;Other;Other | -1.602270 | 0.5976317 | -2.681032 | 0.0278814 | 0.8122951 |
| k__Bacteria;p__Proteobacteria;c__Gammaproteobacteria;o__Pseudomonadales;f__Moraxellaceae;g__Acinetobacter;Other | 1.274251 | 0.4807659 | 2.650461 | 0.0292349 | 0.8122951 |
| k__Bacteria;p__Firmicutes;c__Bacilli;o__Lactobacillales;f__Enterococcaceae;g**Enterococcus;s** | -0.805053 | 0.3125761 | -2.575542 | 0.0328432 | 0.8122951 |
| k__Bacteria;p__Firmicutes;c__Clostridia;o__Clostridiales;f__Clostridiaceae;Other | 1.446479 | 0.5660630 | 2.555333 | 0.0338922 | 0.8122951 |
| k__Bacteria;p__Firmicutes;c__Clostridia;o__Clostridiales;f__Clostridiaceae;Other;Other | 1.450740 | 0.5681802 | 2.553310 | 0.0339991 | 0.8122951 |
| k__Bacteria;p__Actinobacteria | -1.159158 | 0.4803493 | -2.413156 | 0.0422994 | 0.8122951 |
| k__Bacteria;p__Proteobacteria;c__Gammaproteobacteria;o__Pseudomonadales;f__Moraxellaceae;g__Acinetobacter | 1.331243 | 0.5766584 | 2.308546 | 0.0498020 | 0.8122951 |

**Supplementary material 8 – Significantly differing abundances of samples of biliary atresia patient with clearance of jaundice *versus* patients without clearance of jaundice during follow-up, 1 week after Kasai portoenterostomy.**

| **Taxon** | **Beta** | **Standard error** | **F** | **P** | **Adjusted P** |
| --- | --- | --- | --- | --- | --- |
| k__Bacteria;p__Fusobacteria;c__Fusobacteriia | 0.4151485 | 0.1288080 | 3.223003 | 0.0145925 | 0.537713 |
| k__Bacteria;p__Fusobacteria | 0.4468075 | 0.1414644 | 3.158446 | 0.0159629 | 0.537713 |
| k__Bacteria;p__Proteobacteria;c__Alphaproteobacteria;o__Caulobacterales | 0.9776091 | 0.3327785 | 2.937717 | 0.0217838 | 0.537713 |
| k__Bacteria;p__Fusobacteria;c__Fusobacteriia;o__Fusobacteriales | 0.3358073 | 0.1143089 | 2.937717 | 0.0217838 | 0.537713 |
| k__Bacteria;p__Proteobacteria;c__Alphaproteobacteria | 0.9159963 | 0.3232144 | 2.834021 | 0.0252601 | 0.537713 |
| k__Bacteria;p__Proteobacteria;c__Alphaproteobacteria;o__Caulobacterales;f__Caulobacteraceae | 0.7979851 | 0.3000012 | 2.659940 | 0.0324700 | 0.537713 |
| k__Bacteria;p__Actinobacteria;c__Actinobacteria;o__Actinomycetales;f__Microbacteriaceae | 1.1725530 | 0.4408194 | 2.659940 | 0.0324700 | 0.537713 |
| k__Bacteria;p__Fusobacteria;c__Fusobacteriia;o__Fusobacteriales;f__Fusobacteriaceae | 0.3207543 | 0.1205871 | 2.659940 | 0.0324700 | 0.537713 |

**Supplementary material 9 – Significantly differing abundances of samples of biliary atresia patient with clearance of jaundice *versus* patients without clearance of jaundice during follow-up, one month after Kasai portoenterostomy.**

| **Taxon** | **Beta** | **Standard error** | **F** | **P** | **Adjusted P** |
| --- | --- | --- | --- | --- | --- |
| k__Bacteria;p__Actinobacteria | -1.3014521 | 0.2820484 | -4.614286 | 0.0024426 | 0.7804641 |
| k__Bacteria;p__Bacteroidetes;c__Bacteroidia;o__Bacteroidales;f__Porphyromonadaceae;g__Parabacteroides;s__distasonis | -0.7482197 | 0.1850739 | -4.042815 | 0.0049153 | 0.780464 |

**Supplementary material 10 – Significantly differing abundances of samples of biliary atresia patient with clearance of jaundice *versus* patients without clearance of jaundice during follow-up, three months after Kasai portoenterostomy.**

| **Taxon** | **Beta** | **Standard error** | **F** | **P** | **Adjusted P** |
| --- | --- | --- | --- | --- | --- |
| k__Bacteria;p__Proteobacteria;c__Alphaproteobacteria;o__Caulobacterales;f__Caulobacteraceae;g__ | -1.7102553 | 0.4949801 | -3.455200 | 0.0106163 | 0.7484243 |
| k__Bacteria;p__Proteobacteria;c__Alphaproteobacteria;o__Caulobacterales;f__Caulobacteraceae | -1.7147566 | 0.4982057 | -3.441864 | 0.0108098 | 0.7484243 |
| k__Bacteria;p__Proteobacteria;c__Alphaproteobacteria;o__Caulobacterales | -1.6733763 | 0.4905309 | -3.411358 | 0.0112670 | 0.7484243 |
| k__Bacteria;p__Firmicutes;c__Erysipelotrichi | -1.3597072 | 0.4004020 | -3.395855 | 0.0115073 | 0.7484243 |
| k__Bacteria;p__Proteobacteria;c__Alphaproteobacteria;o__Caulobacterales;f__Caulobacteraceae;g**;s** | -1.6234883 | 0.4950498 | -3.279445 | 0.0134974 | 0.7484243 |
| k__Bacteria;p__Firmicutes;c__Erysipelotrichi;o__Erysipelotrichales | -1.3453024 | 0.4286656 | -3.138350 | 0.0164170 | 0.7484243 |
| k__Bacteria;p__Actinobacteria;c__Actinobacteria;o__Actinomycetales;f__Micrococcaceae;g**Rothia;s** | 0.8672579 | 0.2832356 | 3.061966 | 0.0182728 | 0.7484243 |
| k__Bacteria;p__Verrucomicrobia | -1.2953233 | 0.4272642 | -3.031668 | 0.0190697 | 0.7484243 |
| k__Bacteria;p__Firmicutes;c__Erysipelotrichi;o__Erysipelotrichales;f__Erysipelotrichaceae | -1.2916047 | 0.4396165 | -2.938026 | 0.0217743 | 0.7484243 |
| k__Bacteria;p__Proteobacteria;c__Gammaproteobacteria;o__Enterobacteriales;f__Enterobacteriaceae;g__Klebsiella | -1.7653477 | 0.6079224 | -2.903903 | 0.0228583 | 0.7484243 |
| k__Bacteria;p__Proteobacteria;c__Gammaproteobacteria;o__Enterobacteriales;f__Enterobacteriaceae;g__Klebsiella;Other | -1.6773920 | 0.5945796 | -2.821140 | 0.0257312 | 0.7484243 |
| k__Bacteria;p__Bacteroidetes;c__Flavobacteriia;o__Flavobacteriales | -1.6182112 | 0.5780780 | -2.799295 | 0.0265511 | 0.7484243 |
| k__Bacteria;p__Proteobacteria;c__Gammaproteobacteria;o__Enterobacteriales;f__Enterobacteriaceae;g**Klebsiella;s** | -1.6640379 | 0.5989760 | -2.778138 | 0.0273715 | 0.7484243 |
| k__Bacteria;p__Firmicutes;c__Clostridia;o__Clostridiales;f__Clostridiaceae;g__Clostridium | 1.2256866 | 0.4671541 | 2.623731 | 0.0342230 | 0.7484243 |
| k__Bacteria;p__Firmicutes;c__Clostridia;o__Clostridiales;f__Veillonellaceae;Other;Other | 1.8209139 | 0.7045958 | 2.584338 | 0.0362425 | 0.7484243 |
| k__Bacteria;p__Firmicutes;c__Clostridia;o__Clostridiales;f__Veillonellaceae;Other | 1.8142335 | 0.7025600 | 2.582318 | 0.0363493 | 0.7484243 |
| k__Bacteria;p__Firmicutes;c__Clostridia;o__Clostridiales;f__Clostridiaceae;g__Clostridium;s__perfringens | 1.2226469 | 0.4738327 | 2.580335 | 0.0364545 | 0.7484243 |
| k__Bacteria;p__Firmicutes;c__Clostridia;o__Clostridiales;f__Clostridiaceae | 1.2021634 | 0.4709856 | 2.552442 | 0.0379680 | 0.7484243 |
| k__Bacteria;p__Bacteroidetes;c__Flavobacteriia;o**Flavobacteriales;f**[Weeksellaceae] | -1.6114815 | 0.6576687 | -2.450294 | 0.0440883 | 0.7484243 |
| k__Bacteria;p__Proteobacteria;c__Gammaproteobacteria;o__Pseudomonadales;f__Moraxellaceae;g__Acinetobacter;s__guillouiae | 0.9722833 | 0.3973017 | 2.447216 | 0.0442877 | 0.7484243 |

**Supplementary material 11 – Significantly differing abundances of samples of biliary atresia patient with clearance of jaundice *versus* patients without clearance of jaundice during follow-up, six months after Kasai portoenterostomy.**

| **Taxon** | **Beta** | **Standard error** | **F** | **P** | **Adjusted P** |
| --- | --- | --- | --- | --- | --- |
| k__Bacteria;p__Firmicutes;c__Clostridia;o__Clostridiales | 0.9710411 | 0.2067795 | 4.696022 | 0.0093359 | 0.7286235 |
| k__Bacteria;p__Firmicutes;c__Clostridia | 1.0244969 | 0.2206599 | 4.642877 | 0.0097131 | 0.7286235 |
| k__Bacteria;p__Proteobacteria;c__Betaproteobacteria;o__Neisseriales;f__Neisseriaceae;g**Neisseria;s** | -4.4901621 | 1.0399248 | -4.317776 | 0.0124693 | 0.7286235 |
| k__Bacteria;p__Firmicutes;c__Clostridia;o__Clostridiales;f__Veillonellaceae | 0.8429318 | 0.2066360 | 4.079307 | 0.0151070 | 0.7286235 |
| k__Bacteria;p__Firmicutes;c__Clostridia;o__Clostridiales;f__Veillonellaceae;g__Veillonella | 0.7573928 | 0.2019617 | 3.750181 | 0.0199439 | 0.7286235 |
| k__Bacteria;p__Proteobacteria;c__Gammaproteobacteria;o__Enterobacteriales;f__Enterobacteriaceae;g__Klebsiella;s__oxytoca | -2.4435896 | 0.6592944 | -3.706371 | 0.0207196 | 0.7286235 |
| k__Bacteria;p__Firmicutes;c__Bacilli;o__Lactobacillales;f__Streptococcaceae;g__Streptococcus | 0.7255641 | 0.2004905 | 3.618945 | 0.0223781 | 0.7286235 |
| k__Bacteria;p__Firmicutes;c__Bacilli;o__Lactobacillales;f__Streptococcaceae | 0.7879192 | 0.2305317 | 3.417834 | 0.0268335 | 0.7286235 |
| k__Bacteria;p__Firmicutes;c__Clostridia;o__Clostridiales;f__Lachnospiraceae;g__Oribacterium | -3.6133367 | 1.0951937 | -3.299267 | 0.0299540 | 0.7286235 |
| k__Bacteria;p__Firmicutes;c__Clostridia;o__Clostridiales;f__Lachnospiraceae;g**Oribacterium;s** | -3.5939188 | 1.0968077 | -3.276708 | 0.0305953 | 0.7286235 |
| k__Bacteria;p__Firmicutes;c__Erysipelotrichi;o__Erysipelotrichales | -0.8449023 | 0.2629205 | -3.213528 | 0.0324797 | 0.7286235 |
| k__Bacteria;p__Proteobacteria;c__Gammaproteobacteria;o__Enterobacteriales;f__Enterobacteriaceae;g__Klebsiella;Other | -1.9241149 | 0.6002882 | -3.205319 | 0.0327345 | 0.7286235 |
| k__Bacteria;p__Firmicutes;c__Erysipelotrichi | -0.8440274 | 0.2880533 | -2.930108 | 0.0428092 | 0.7286235 |
| k__Bacteria;p__Firmicutes;c__Erysipelotrichi;o__Erysipelotrichales;f__Erysipelotrichaceae | -0.8470234 | 0.2910150 | -2.910584 | 0.0436526 | 0.7286235 |
| k__Bacteria;p__Bacteroidetes;c__Bacteroidia;o__Bacteroidales;f__Bacteroidaceae | -0.7210838 | 0.2532142 | -2.847723 | 0.0465028 | 0.7286235 |
